# Antibody-Dependent Enhancement and Zika: Real Threat or Phantom Menace?

**DOI:** 10.3389/fcimb.2018.00044

**Published:** 2018-02-15

**Authors:** Miguel A. Martín-Acebes, Juan-Carlos Saiz, Nereida Jiménez de Oya

**Affiliations:** Department of Biotechnology, Instituto Nacional de Investigación y Tecnología Agraria y Alimentaria, Madrid, Spain

**Keywords:** Zika virus, dengue virus, West Nile virus, antibody-dependent enhancement (ADE), vaccines

## Introduction

Host-pathogen interaction between Zika virus (ZIKV) and other medically relevant flaviviruses is a hot topic, in part due to the potential risk of disease exacerbation by antibody-dependent enhancement (ADE). Current data are controversial, thus care should be taken when facing this aspect of ZIKV infection, particularly during vaccine development.

## Zika virus epidemic

Mosquito-borne flaviviruses are (re-)emerging pathogens responsible for several human diseases that lately have been raising alarms both socially and in healthcare. Colonization of new geographical areas by vectors and spread of ZIKV to regions with competent vectors allows its increased settling, exemplified by the recent epidemic across new areas where other important flaviviruses, such as dengue virus (DENV), Yellow Fever virus (YFV) and West Nile virus (WNV), co-circulate. The alarm was mainly due to the rapid spread of the virus across the American continent (Ali et al., 2017; Zhang et al., 2017). Previously, ZIKV infection was associated with mild, flu-like symptoms, but currently several serious neurological complications including Guillain-Barré syndrome and fetal/neonatal microcephaly have been directly linked to it. In this scenario, the viability of a vaccine against ZIKV as a preventive strategy is gaining force (Saiz et al., 2017), supported by previous experiences with flavivirus vaccines such as that against YFV. However, development of flaviviral immunity may also carry disadvantages that must be taken into account before undertaking massive vaccination campaigns.

## Antibody-dependent enhancement of infection

**Figure 1:**
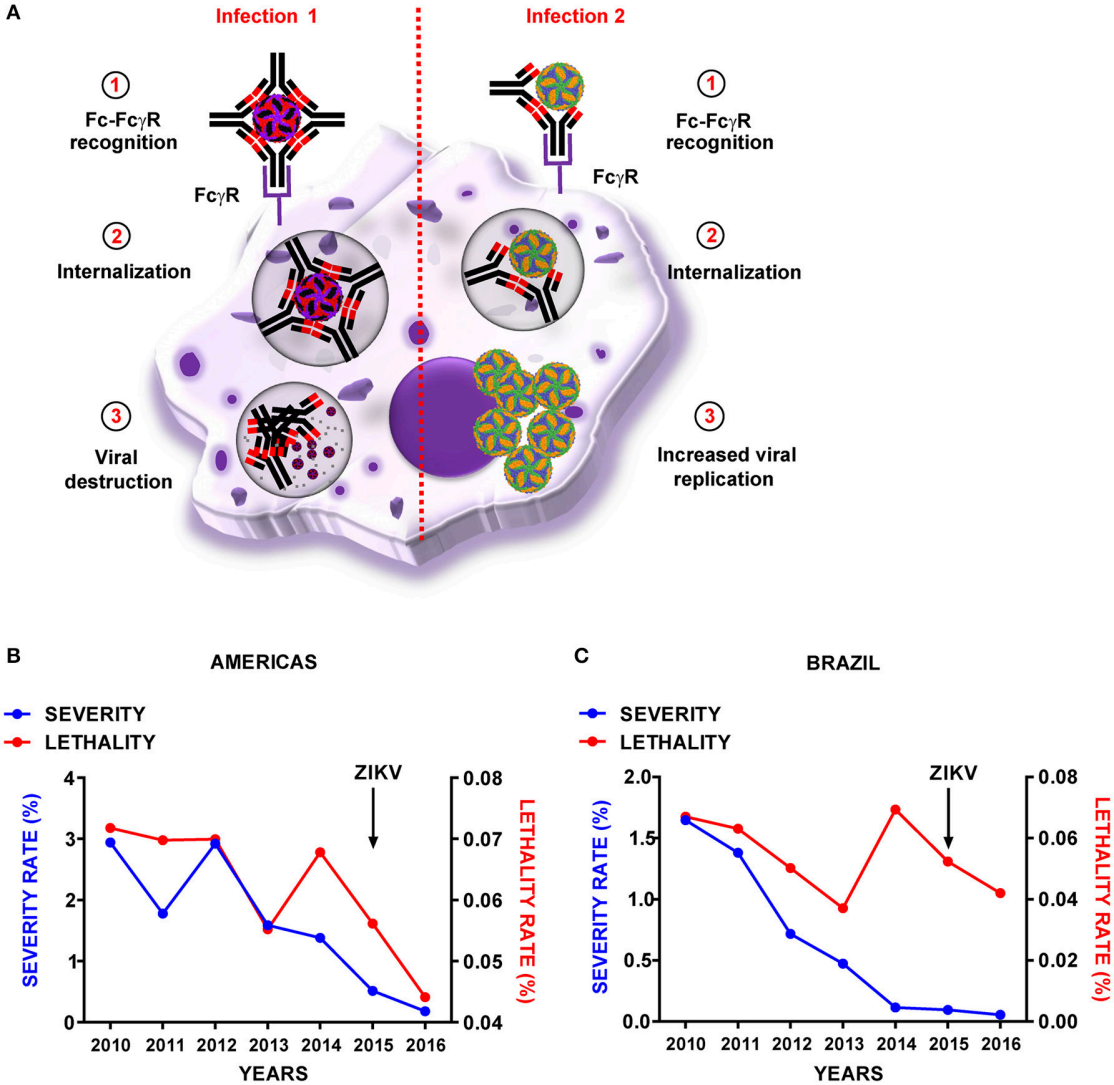
Representation of dengue virus antibody-dependent enhancement and reported cases in the Americas. **(A)** Schematic representation of dengue virus antibody-dependent enhancement phenomenon. Graphical representation of DENV-severity and-lethality rates across the years in the Americas **(B)** and Brazil **(C)** Zika virus (ZIKV) epidemic is indicated with an arrow. Dengue severity was calculated as the number of cases including severe dengue and hemorrhagic dengue fever/total dengue cases. Lethality rate was calculated as the number of dengue-associated deaths/total dengue cases. Data were obtained from the Pan American Health Organization/World Health Organization (PAHO/WHO) website: (http://www.paho.org/hq/index.php?option=com_topics&view=readall&cid=3273&Itemid=40734&lang=en).

Flaviviruses often show antigenic cross-reactivity, with shared immunogenic epitopes for stimulation of both humoral and cell-mediated immune responses. This cross-reactivity can be beneficial and result in cross-protection; however, humoral cross-reactivity can also exacerbate disease by the phenomenon of antibody-dependent enhancement (ADE) (Halstead, 2014), of which DENV is the prototypic model. Cells with phagocytic activity and bearing Fc receptors (FcR) are able to help to the clearance of pathogens coated with antibodies. The process implies the recognition of the constant portion of the antibody by the receptor in a class-dependent manner and greatly improves phagocytic activity of cells but also can become a type of immunopathology when is “exploited” by pathogens as DENV. Primary DENV infection results in a mild, acute disease with production of efficient neutralizing antibodies, in which virus-antibody complexes are recognized by FcR, internalized and destroyed. Problems may arise when a second DENV infection of a different serotype occurs, as the antibodies produced during the first infection can recognize and bind the second infecting strain, but with sub-neutralizing capability (Halstead, 2014). Thus, cells bearing FcR uptake and internalize antibody-coated viruses that are able to replicate within (Figure 1A). This phenomenon can also enable the virus to infect non-permissive cells, and has been related to lower antiviral responses. Severe disease outcome is therefore related not only to early high viremia, but also to lower levels of innate immune mediators such as nitric oxide (NO) or interferon (IFN) transcripts, and to a higher production of interleukines such as IL-10 (Halstead, 2014). Although recent studies also support an increased risk of developing severe dengue disease in humans with pre-existing anti-DENV antibodies (Katzelnick et al., 2017), the controversy persists, because cases of dengue hemorrhagic fever have been reported in primary infections by dengue (Khurram et al., 2014; Halstead and Cohen, 2015; Soo et al., 2016). Dengue ADE has been linked to the production of cross-reactive antibodies against the precursor-membrane protein (prM) of the DENV viral surface, which might increase the infectivity of immature virions carrying high amounts of uncleaved prM (Dejnirattisai et al., 2010). Apart from data obtained *in vitro*, ADE activity has not been described for other flaviviruses. However, due to the potential disastrous consequences of ADE between ZIKV and other flaviviruses, this possibility must be kept in mind.

## Potential for ADE between zika and other related viruses

A prerequisite for ADE to occur is the existence of antigenic cross-reactivity. Antibodies from DENV- or ZIKV-infected humans (Dejnirattisai et al., 2016; Paul et al., 2016; Sapparapu et al., 2016; Stettler et al., 2016; Kam et al., 2017) and non-human primates (Keasey et al., 2017; Pantoja et al., 2017), and from WNV-infected patients (Bardina et al., 2017) have shown a wide variety of *in vitro* cross-reactivity, with a primary tendency toward high cross-reactivity, especially between ZIKV and DENV. In other cases, highly specific or no cross-reactive antibodies against ZIKV or WNV have been observed (Stettler et al., 2016; Keasey et al., 2017; Vázquez-Calvo et al., 2017). Even cross-reactive antibodies can be protective if neutralization of heterotypic virus still occurs; however, *in vitro* seroneutralization assays have shown an absent or poor neutralizing capability for the majority of ZIKV and DENV cross-reactive antibodies tested, with few inducing *in vitro* cross-neutralization (Dejnirattisai et al., 2016; Kawiecki and Christofferson, 2016; Paul et al., 2016; Stettler et al., 2016; Bardina et al., 2017; Kam et al., 2017). Seroneutralization between WNV- and ZIKV-specific antibodies was also lacking (Bardina et al., 2017; Vázquez-Calvo et al., 2017). In contrast, the vast majority of DENV- and ZIKV-specific antibodies tested were able to increase heterotypic viral replication in cell culture (Dejnirattisai et al., 2016; Kawiecki and Christofferson, 2016; Paul et al., 2016; Bardina et al., 2017; Kam et al., 2017; Londono-Renteria et al., 2017). This has been postulated to be an ADE-mediated phenomenon, as it was prevented by abolishment of Fc-Fc_γ_R interaction by antibody mutation (Stettler et al., 2016) or pre-treatment with α-FcR antibodies (Paul et al., 2016). Similarly, WNV-specific antibodies also enhanced ZIKV infection (Bardina et al., 2017). Although these results agree with previous experimentally-described viral enhancement upon heterotypic flavivirus infection in cultured cells (Fagbami et al., 1987), they are difficult to extrapolate *in vivo*; in fact, animal studies have shown disparate results. A significant increase in mortality was described in ZIKV-infected Stat2^−/−^ mice, with altered IFN responses and permissivity to ZIKV and DENV infections when pretreated with DENV-immune plasma (Bardina et al., 2017). In addition, ZIKV-specific antibodies delivery in AG129 immunocompromised mice resulted in an enhancement of DENV infection (Stettler et al., 2016). Similar results were observed in non-human primates (Rhesus macaques) commonly used as models of dengue disease (George et al., 2017). In contrast, IFNAR^−/−^ mice pre-treated with anti-DENV antibodies were protected against a lethal ZIKV challenge (Kam et al., 2017), nor was ADE observed in 129Sv/ev immunocompetent mice pre-treated with anti-DENV antibodies and challenged with ZIKV (Stettler et al., 2016), or in natural models of ZIKV infection (Rhesus macaques) previously exposed to DENV (McCracken et al., 2017; Pantoja et al., 2017) or YFV (McCracken et al., 2017). Likewise, ZIKV infection protect immunocompetent mice against WNV challenge (Vázquez-Calvo et al., 2017). The difference between ADE and cross-protection could be a product of the varied type and dose of the antibody/immunization route and scheme used. Indeed, in Stat2^−/−^ mice, high concentrations of DENV-immune plasma protected against ZIKV infection, as did pretreatment with WNV-specific antibodies (Bardina et al., 2017). It is worth noting that ZIKV and DENV share immunodominant epitopes that elicited cross-reactive T cells (Grifoni et al., 2017; Wen et al., 2017) with *in vivo* protective roles (Wen et al., 2017), which could also explain the differences observed between experiments using plasma administration or virus immunization.

## Current epidemiological scenario

Regarding the current data on the clinical relevance of ADE for ZIKV infection, ZIKV has co-circulated with DENV and other flaviviruses in several countries with high rates of seroprevalence, and to-date, no change in clinical outcome has been correlated with the presence of other flaviviruses. During an outbreak in French Polynesia (2008), ZIKV infection was linked to Guillain-Barré development independent of previous DENV infection (Cao-Lormeau et al., 2016). Other neurological complications, such as microcephaly, have also been related to ZIKV infection (Martines et al., 2016). The hypothesis of ADE behind ZIKV-related microcephaly has been raised by expertise in the field, but remains to be explored (Miner and Diamond, 2017). However, a recent work, has shown that changes in the virus, specifically a mutation in the prM protein, will be more likely responsible for the development of microcephaly (Yuan et al., 2017) which could explain the increased rate during the last epidemic in the Americas, since evolutionary analysis indicated that this substitution was not present in previous ancestors. Additionally, the ZIKV epidemic in the Americas, where DENV is a very important concern, has not altered the downward trend in severity and lethality of dengue (Figures 1B,C). However, these data should be taken with caution, because they are based on clinical reported cases and not all of them have been assayed for laboratory confirmation. Nevertheless, they point to a lack of DENV-infection enhancement due to ZIKV circulation. Accordingly, recent epidemiological surveys have showed that patients with prior DENV infection exhibited no signs correlated with ADE (as increased viral loads or pro-inflammatory cytokine profiles) in subsequent ZIKV infections (Bernardes-Terzian et al., 2017). Additionally, epidemiologic data suggests that vaccination against YFV could be related with protection against ZIKV (De Góes Cavalcanti et al., 2016), which further supports the lack of ADE among ZIKV and other flaviviruses. Nonetheless, more efforts, ideally with adequate immunocompetent animal models, must be performed to answer the questions about ZIKV and ADE. It is expected that, together with seroepidemiological surveillance data, these studies will contribute to a better understanding of the interplay between flaviviruses and the risk-to-reward ratio of ZIKV vaccines.

## Author contributions

All authors listed, have made substantial, direct and intellectual contribution to the work, and approved it for publication.

### Conflict of interest statement

The authors declare that the research was conducted in the absence of any commercial or financial relationships that could be construed as a potential conflict of interest.
